# Screen Printed Passives and Interconnects on Bio-Degradable Medical Hydrocolloid Dressing for Wearable Sensors

**DOI:** 10.1038/s41598-019-53033-4

**Published:** 2019-11-25

**Authors:** Haneen Alsuradi, Jerald Yoo

**Affiliations:** 1 Department of Electrical and Computer Engineering, National University of Singapore (NUS), Singapore, Singapore; 2 Department of Electrical Engineering and Computer Science (EECS), Khalifa University of Science and Technology, Abu Dhabi, UAE; 3 Singapore Institute for Neurotechnology (SINAPSE), Singapore, Singapore

**Keywords:** Electrical and electronic engineering, Electronic and spintronic devices

## Abstract

The healthcare system is undergoing a noticeable transformation from a *reactive, post-disease treatment* to a *preventive, predictive continuous healthcare*. The key enabler for such a system is a pervasive wearable platform. Several technologies have been suggested and implemented as a wearable platform, but these technologies either lack reliability, manufacturing practicability or pervasiveness. We propose a screen-printed circuit board on bio-degradable hydrocolloid dressings, which are medically used and approved, as a platform for wearable biomedical sensors to overcome the aforementioned problems. We experimentally characterize and prepare the surface of the hydrocolloid and demonstrate high-quality screen-printed passive elements and interconnects on its surface using conductive silver paste. We also propose appropriate models of the thick-film screen-printed passives, validated through measurements and FEM simulations. We further elucidate on the usage of the hydrocolloid dressing by prototyping a Wireless Power Transfer (WPT) sensor and a humidity sensor using printed spiral inductors and interdigital capacitors, respectively.

## Introduction

Wearable healthcare or ambulatory monitoring for pervasive, preventive healthcare requires pervasive, personalized wearable sensors. Conventional methods rely on bulky machines and cumbersome wires combined with wet electrodes. This type of monitoring makes it laborious for patients to live their daily routine normally during the monitoring session. Additionally, monitoring a patient at a specific time may not necessarily reflect his true medical condition^1,2^. Therefore, it is necessary to have a wearable, non-wired, comfortable technology that will allow the seamless, continuous monitoring without limiting the patient’s mobility. The pervasive wearable platform will make it possible to monitor the body condition by sensing physiological signals and if needed, proactively respond to them.

**Figure 1:**
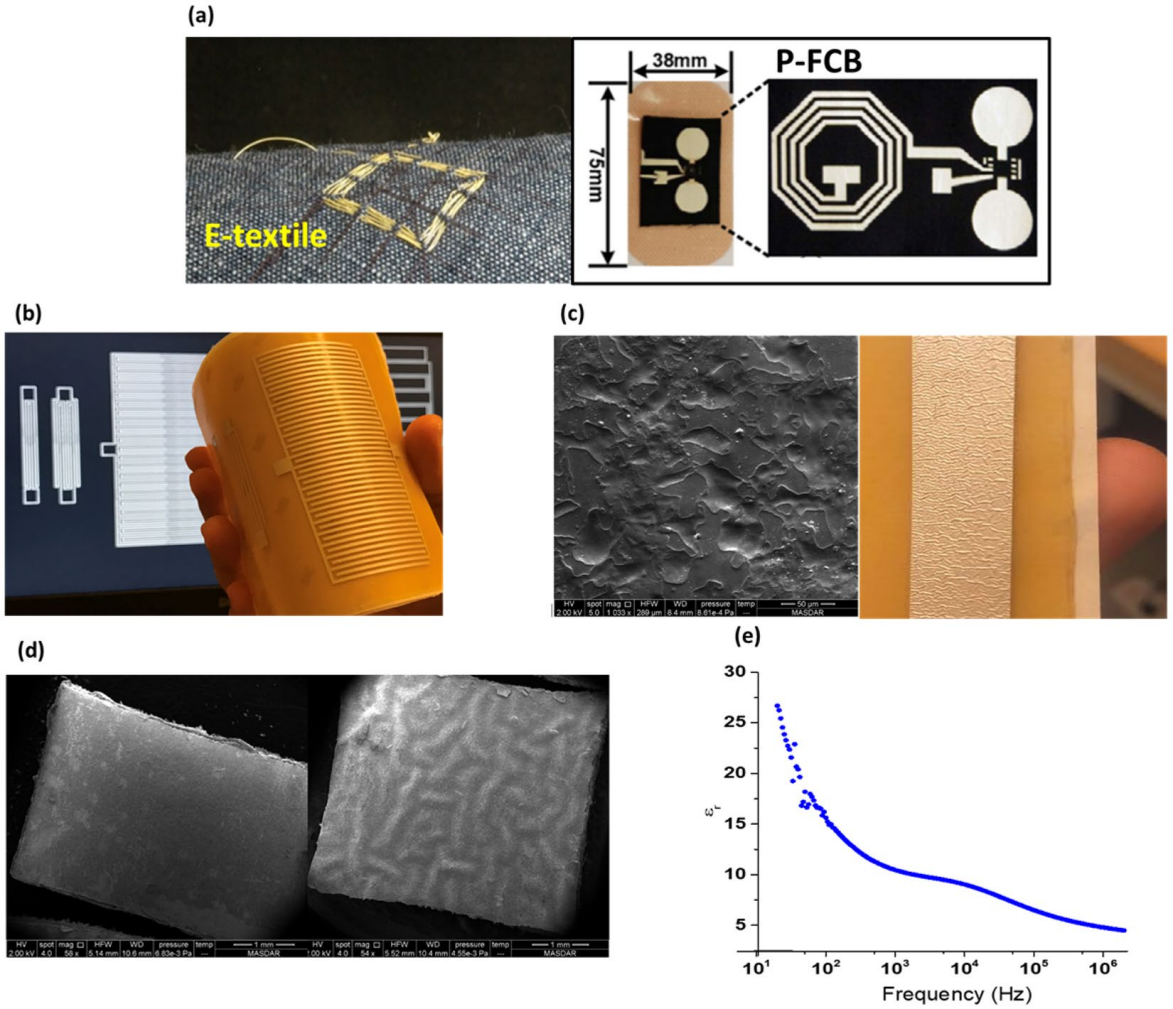
(**a**) Conventional flexible substrates (the figure to the left is by J. Yoo *et al*., JSSC 2009, Nov.^15^ and to the right is by J. Yoo *et al*., IEEE 2009^16^) (**b**) Proposed hydrocolloid dressing substrate (**c**) Non-sticky surface of the hydrocolloid under SEM. A stripe of silver paste is applied on the surface showing non-smooth distribution (**d**) Silver covered hydrocolloid piece before (right) and after (left) surface preparation under the SEM (the figure is by H. Alsuradi *et al*., ISCAS 2017, May^17^) (**e**) Dielectric constant vs. frequency of 3 M Tegaderm hydrocolloid.

With advancements in the fields of electronics and materials science and their tools, a whole new door is opened to professionally integrate electronics into clothing and flexible substrates. Substantial work has been done in the area of wearable electronics and e-textiles in the past decade, specifically for healthcare applications. Wearable Electronics can be categorized into three categories. The first is Flexible Printed Circuit Board (FPCB) in which flexible plastic is used as its substrate^3–7^. The substrate is attached to a garment, but since its hardness and thermal coefficient are different than those of the garment, the wearer will feel obtrusiveness and stiffness; moreover, the substrate is not bio-degradable. The second category is E-textiles, in which conductive threads are used as interconnects^8–10^. This technique is more comfortable for the user, but threads are more susceptible to cuts and faults; also, threads must be stitched during the garment manufacturing process, which is not mass production friendly. The third category is the Planar-Fashionable Circuit Board (P-FCB) in which planar passives and interconnects are screen-printed on fabric and integrated with printed electrodes and ICs^11–14^. This method is more pervasive than the aforementioned technologies as it exploits the garment itself as the substrate. However, only particular types of fabric can be used for successful printing; for example, the fabric should be dense, and fibers should be fine enough to reduce the resistance and to maintain the integrity of the printed connections. Moreover, P-FCB electrodes do not stick to the body well, which makes it susceptible to motion artifacts. Fig. 1a shows the e-textile^15^ and the P-FCB^16^ wearable electronics technologies (fabricated and photo taken by the authors).

In this work, we propose the bio-degradable medical hydrocolloid dressings as a platform for screen-printed passives and interconnects and the integration of ICs, to completely replace the conventional hard printed circuit boards (PCBs). Medical hydrocolloids are flexible dressings that are medically used and approved. A hydrocolloid has a self-adhering surface on one side and a water-resistant surface on the other side, allowing an extended time of usage up to 7 days. Hydrocolloids come in different shapes, sizes and thicknesses. We physically and electrically characterize a hydrocolloid and prepare its surface before printing. A medical hydrocolloid with an Interdigital Capacitator (IDC) screen-printed on its surface is shown in Fig. 1b (photo taken in the authors’ laboratory). We also verify the capability of hydrocolloids serving as substrates by designing, fabricating and characterizing printed passives (L, C, R) and integrating them in applications such as Wireless Power Transfer (WPT) and smart adhesive bandage. Appropriate mathematical models are selected and modified to aid in the design process of the printed passives^17–21^. The proposed hydrocolloid dressing platform solves the problems above of the current wearable technologies: it offers an excellent replacement of stiff or unreliable substrates without adding restrictions on the wearer’s clothing. It is patient-friendly and practicable in the circuit fabrication process. The shape and the size of the hydrocolloids have negligible effects on the fabricated passives.

## Results

### Physical and electrical characteristics of the hydrocolloid dressing

Before printing, it is critical to investigate and analyze the physical properties of the hydrocolloid substrate including its smoothness and thickness. Information about smoothness shows whether any surface preparation is needed, while the thickness of the substrate is required for accurate passives design. In this study, hydrocolloid dressings from 3 M (Tegaderm^TM^) with planar dimensions of 10 cm × 10 cm is selected. Fig. 1c shows an SEM micrograph of the printing surface, which is the water-resistant non-sticky side of the dressing. It shows that the surface is rough with uneven micro-features in which their dimensions are comparable with the patterns appearing after applying the silver paste, shown in Fig. 1c; to print a robust and an accurate passive on it, we apply a transparent layer of Polyvinyl Acetate (PVA) with few microns thickness to fill in the pores and thus to smoothen the surface. Fig. 1d which was presented in our previous work^17^ shows the SEM image before and after the smoothening. The substrate’s electrical permittivity, measured as a function of frequency, is shown in Fig. 1e. It can be noticed that the permittivity declines as the frequency increases. The decline can be explained by the different processes involved at the microscopic level (ionic and dipolar relaxation) in determining the value of the complex permittivity and thus indicating the amount of energy lost and stored. This measurement essential for designing screen-printed inter-digital capacitors. The dielectric constant is around 4.7 at 2 MHz.

### Printed passives fabrication and characterization

**Figure 2:**
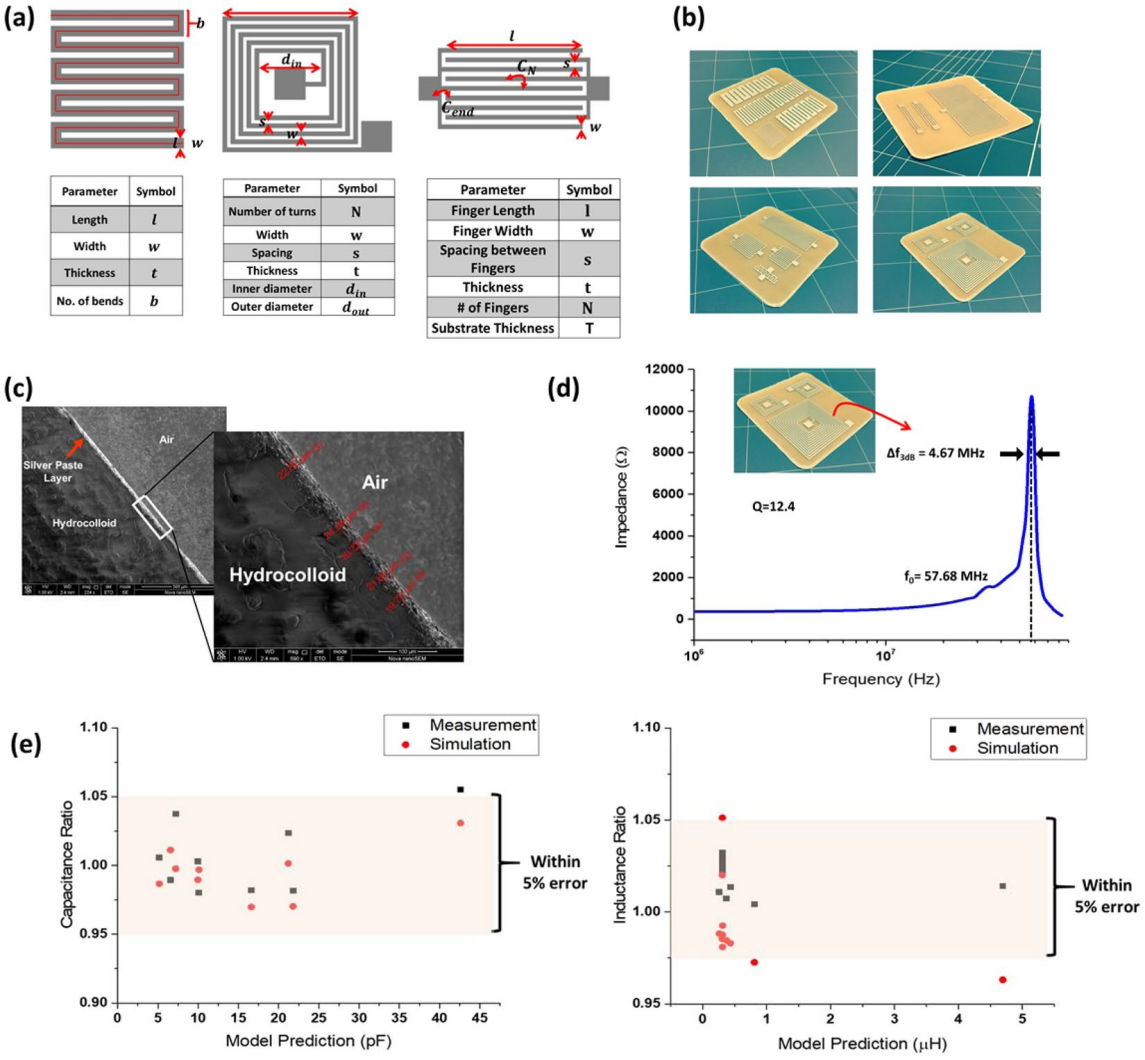
(**a**) Layout and design parameters of screen-printed LCR (**b**) A sample of the fabricated passives on hydrocolloids (**c**) Cross-sectional micrograph showing the thickness of the metallization layer after screen-printing passives (**d**) Self Resonance Frequency measurement of an N = 11 fabricated square spiral (**e**) Comparison between measurements and simulations vs the selected models prediction for fabricated IDCs and spirals in the form of ratios.

We use screen-printing as a fabrication method because it is a thick-film technology leading to low resistance traces and interconnects, and most importantly, is mass-production friendly. Serpentine resistors, Interdigital capacitors (IDC) and spiral square inductors were designed and screen-printed onto the smoothened hydrocolloid’s surface. The general layout of the three passives and their design parameters are shown in Fig. 2a. Designs were created such that extreme cases of each design parameter were selected while considering the area limitation of the utilized hydrocolloid. Designs were created on a CAD tool and exposed on a mesh for printing. Hydrocolloids were smoothened by applying a transparent layer of Polyvinyl Acetate (PVA) to fill the micro-features. Conductive silver paste (Paron90) was utilized for screen-printing and Fig. 2b shows a sample of the fabricated passives. The thickness of metallization was measured by imaging a cross section of a screen-printed sample. Fig. 2c shows an SEM cross-sectional micrograph showing a metallization thickness of around 20 µm. This thickness can be mainly controlled by the mesh size or the number of screen-printing iterations.

Fabricated samples were characterized: resistance was measured for serpentine resistors, the capacitance for IDCs and inductance for spirals. These measurements were compared against FEM simulations and mathematical models’ prediction for the same passives’ designs considering the hydrocolloids properties. Fig. 2e shows the measurement comparison of fabricated IDCs and spirals. Measurements and simulations values are reported in the form of ratios with respect to the predicted values by the proposed mathematical model. Each of the measurements was replicated three times at 2 MHz, which is sufficiently below their self-resonance frequencies (SRF). As an example, SRF of a large inductor with *N* = 11 was measured to be around 58 MHz as shown in Fig. 2d.

**Figure 3:**
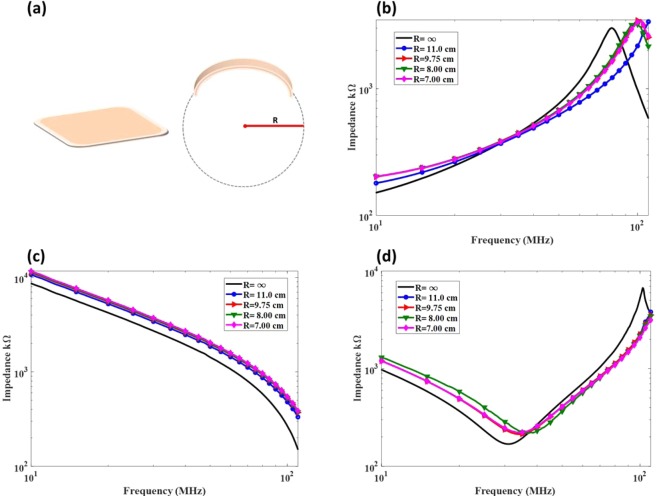
(**a**) Illustration of hydrocolloid before and after mechanincal stress (bending) where R is the bending parameter (**b**) Impedance curve of a fabricated inductor under mechanical stress (**c**) Impedance curve of a fabricated capacitor under mechanical stress (**d**) Impedance curve of a fabricated LC tank under mechanical stress.

Reliability tests were also performed on the printed passives by assessing their impedance while mechanically deformed. This test is crucial for confirming the reliability of the fabricated wearable electronics as it resembles a realistic wearable environment where convexity is present. The test was applied on three different passives (one spiral inductor, one interdigitated capacitor and one LC tank). The bending parameter, denoted herein as R, is the radius of a virtual circle created by extending the arc which is formed by the bandage convexity as depicted in Fig. 3a. Flat bandage (no convexity) has R = infinity and more convexity is indicated by a smaller R. The impedance change in the passives with respect to the change in R is shown in Fig. 3b–d for a bandage of inductor, capacitor and an LC tank, respectively. It can be seen that the pattern and behavior of the passive is conserved under the mechanical stress with a minor difference in the impedance measurement. Additionally, the measurement highlights the resonance frequencies of the inductor and LC tank; however, the resonance frequency of the capacitor is beyond the capability of the utilized instrument.

### Printed passives modeling and tuning

Layouts of passives shown in Fig. 2a are readily borrowed from passives printed on printed circuit boards (PCBs) in the field of microwave integrated circuits (MICs). Several models in the literature describe those passives (resistance R, capacitance C, and inductance L) in the domain of PCB and thin-film fabrication processes and it was of a target to validate if those models can be straightforwardly applied to screen-printed passives on hydrocolloids. Screen-printing usually yields structures with thicker metallic films as compared to other thin-film methods like sputtering. Both measurements and FEM simulations were utilized as validators for the best model that can be used for our purpose.

#### Resistance

Serpentine resistors are easily modeled using their design parameters by the Eq. (1) below:

1 $$R=\frac{l-(bw)}{\sigma \,w\,t}$$

where *l*, *b*, *w* and *t* are length, number of bends, width and, thickness respectively.

#### Capacitance

In screen printing technology, *l* and *w* are in the range of mm while *t* is in µm. Thickness of the substrate can greatly affect the capacitance. The capacitance increases with the substrate thickness; however, in case the substrate *t* is much greater than the finger *w* of the IDC, its capacitance saturates. Capacitance of IDCs is due to the adjacent interdigitated fingers (*C*_*N*_) and due to the fringing fields at the end of each finger (*C*_*end*_), as shown in Fig. 2a. The first model in literature that described IDCs is by Alley *et al*.^18^ which is insensitive for cases where *w* ≠ *s*, while Bahl *el al*.’s model^22^ always overestimates the capacitance value according to simulations. Igreja-Dias *et al*.^19^ and Gevorgian *et al*.^23^ models were found to underestimate capacitance value though the latter considers all capacitive effects. Our proposed capacitance model for IDCs on hydrocolloids is based on the model in^19^ for calculating *C*_*N*_, but additionally considering two very important effects: the non-negligible thickness (*t*) of the screen-printed IDC and the capacitance due to the fringing fields at the end of each finger (*C*_*end*_)^24^. The finite thickness *t* is taken into account by introducing the effective width in *C*_*N*_:

2 $${w}_{eff}=w+\frac{t}{\pi }[1+\,\mathrm{ln}(\frac{4\pi w}{t})]$$

The proposed model is now a function of *C*_*N*_ and *C*_*end*_ and is described by:

3 $${C}_{proposed}={C}_{N}(N,{w}_{eff},s,l,{{\epsilon }}_{r},)+N{C}_{end}$$

Table 1 summarizes the accuracy of those models with respect to FEM simulations regarding the Mean Percentage Error (MPE).

**Table 1:** MPE of IDC models compared to FEM simulations.

Model	MPE
Alley	24.7 %
Bahl	8.01 %
Gevorgian	26.2 %
Igreja-Dias	16 %
Proposed model	4.3%

#### Inductance

Three models describing square spiral inductors have been compared to FEM simulations considering hydrocolloid substrate. Table 2 summarizes the performance of the three models concerning the wide range of simulated spirals. Corrected Current Sheet (CCS) model shows the best accuracy with Mean Percentage Error (MPE) of around 6%. The quadratic dependence of inductance on *N* was found to overestimate the inductance considering the layout shown in Fig. 2a; this is because the contribution of the first turn is always incomplete. The proposed model suggests utilizing the CCS model while changing *N*^2^ dependence to *N*^1.97^.

4 $${L}_{Proposed}=\frac{\mu {N}^{1.97}{d}_{avg}\,{c}_{1}}{2}[\mathrm{ln}(\frac{{c}_{2}}{\rho })+{c}_{3}\rho +{c}_{4}{\rho }^{2}]+{L}_{correction}$$

5 $${L}_{correction}=\frac{\mu {N}^{2}{d}_{avg}\,{c}_{1}}{2}[\frac{0.178(n-1)s}{nl}+0.0833\frac{(n-1)s(s+w)}{{l}^{2}}-\frac{1}{n}\,\mathrm{ln}(\frac{w+t}{w}\,)]$$

where *d*_*avg*_ and *ρ* are the average diameter and fill ratio respectively, *L*_*correction*_ is a correction term to account for the finite thickness of the spiral and *c*_1_, *c*_2_, *c*_3_ and *c*_4_ are constants equal to 1.27, 2.07, 0.18, 0.13 respectively^20^.

**Table 2:** MPE of spiral models compared to FEM simulations.

Model	MPE
Modified Wheeler	8.9 %
Bryan Model	21.8 %
Corrected Current Sheet	5.9 %
Proposed Model	2.7 %

### Wireless power transfer using printed spirals on hydrocolloid

**Figure 4:**
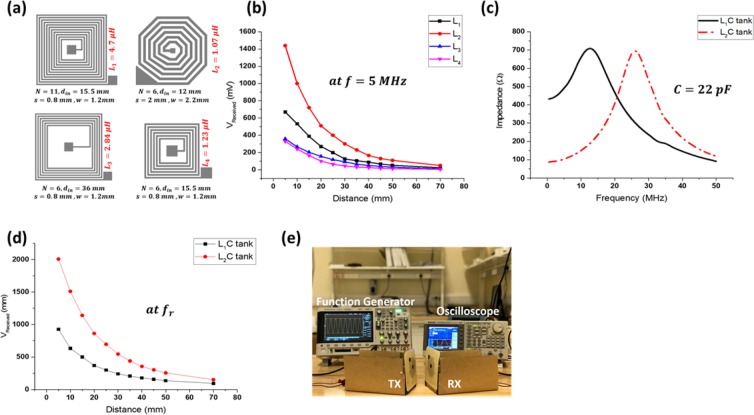
WPT inductors’ design and measurements (**a**) Layout and design parameters of the fabricated spiral inductor pairs (**b**) Received voltage vs. the operation distance at the RX inductor (**c**) LC tanks resonance frequencies (**d**) Received voltage at the RX LC tanks (**e**) Measurement setup of the WPT tests. The tilt between TX and RX is for demonstration purposes. (This figure is by H. Alsuradi *et al*., ISCAS 2017, May^17^).

The design and characterization of printed spirals on hydrocolloid have been thoroughly explained in our previous work and summarized in Fig. 4 ^17^. Here, we highlight the most important findings of our previous work on wireless power transfer of printed spirals on hydrocolloid dressings. Four different pairs of spirals have been designed using Eq. 4 and screen printed on hydrocolloids. The details of spirals designs are shown in Fig. 4a including their design parameters, labels, and inductance value. The spiral pairs have been tested to wirelessly transfer power for wearable applications requiring coupling in cm range. Fabricated pairs have been tested under the inductive coupling mechanism using a carrier of *f* = 5 *MHz* and *V*_*pp*_ = 5 *V*. For each of the pair, the TX and RX inductors were tested while being in an exactly parallel position. It can be observed from Fig. 4b that the octagonal inductor exhibits the best performance with the efficiency of around 20% at 5 mm distance. For square inductors, the higher the value of the inductance the better the performance. To further improve the performance, resonance mechanism has been examined in which the inductor is attached to a capacitor forming an LC tank with a controlled operating frequency. A 22 pF capacitor in hydrocolloid was selected and attached to both *L*_1_ and *L*_2_. The resonance frequency was experimentally measured for both tanks as shown in Fig. 4c. The measurement shows that the octagonal inductor has a lower resonance frequency and a sharper peak indicating a higher Q-factor. It is shown in Fig. 4d that repeating the power transfer experiment at a power carrier of *V*_*pp*_ = 5 *V* boosted the performance of both inductors, *L*_1_ to 20% while *L*_2_ to 40%. Fig. 4e shows the experimental setup that was used while conducting the WPT measurements.

### Smart adhesive bandage using printed IDC on hydrocolloids

**Figure 5:**
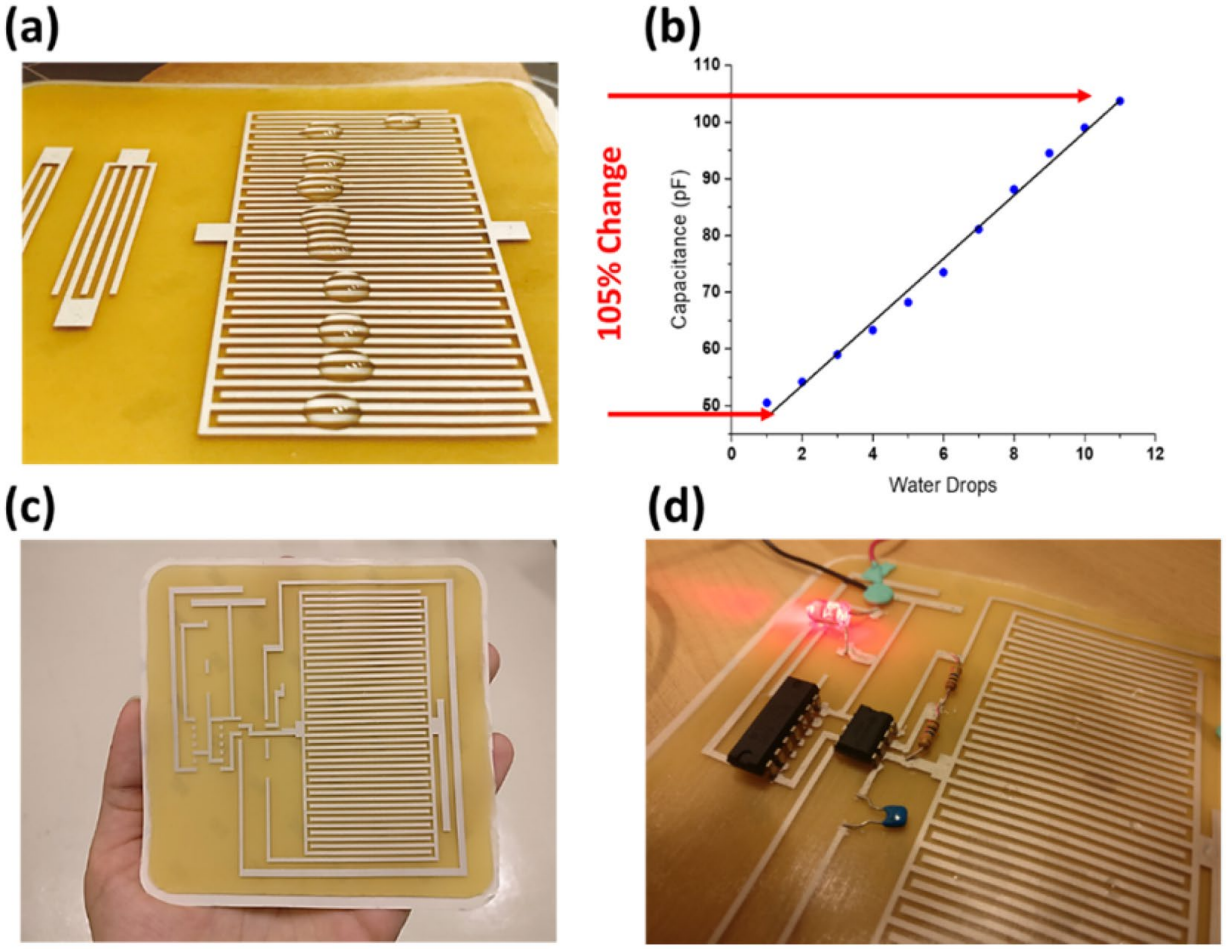
(**a**) Water drops on top of the 43 fingers IDC (**b**)Capacitance change during humidity test on IDC of N = 43 (**c**) Screen-printed IDC, traces and pinholes for the smart bandage circuitry (**d**) Smart bandage while in operation.

We have also demonstrated the possibility of fabricating a smart bandage that is capable of detecting the level of humidity for wet wounds. A 43 fingers IDC was screen-printed and tested as a sensing element for humidity; Fig. 5a. Humidity was controlled by adding drops of water on the surface of the IDC and measuring the capacitance value after each droplet. The nominal capacitance value was measured to be around 44.9 pF, and each droplet contributed ~4–5 pF additional capacitance. The accumulation of water drops on the top of the printed IDC alter the effective permittivity and thus, capacitance changes by up to 105% for 11 drops of water as shown in Fig. 5b. The impedance change is also due to the change in the fingers conductance attributed to the ionic conduction, however in our application, we are interested in the capacitance change solely^25^. This screen-printed sensor was integrated with off-the-shelf components such as resistors, capacitors, a 555-timer, and a counter to form a complete circuitry for humidity sensing. The sensed capacitance is connected to a 555-timer taking part in controlling the output frequency of the timer. This frequency is then divided using an 8-bit counter reducing the frequency for easy visualization and hence, an LED can be used. The higher the humidity, the higher the capacitance and hence the less the output frequency and the less the flashing frequency. Connection points and traces were carefully designed on AutoCAD considering the dimensions of the ICs and their pins. The design was screen-printed, and off-the-shelf components were attached using the same silver paste used in printing. Fig. 5c shows the screen-printed design before placing components. When water drops are placed on the surface of the IDC, we can observe the frequency changes accordingly. For high levels of humidity, the LED saturates to ON state. Fig. 5d shows the smart adhesive bandage while in operation.

## Discussion

### Parameter space of the selected mathematical models

Parameters space defines the range of values for each design parameter for which a particular mathematical model is valid. Parameters space can be bound due to some assumptions the model is built on or due to physical limitations. In the context of passives printed on hydrocolloids, physical limitations can be due to the screen printing technology and the size of the hydrocolloid itself. The Minimum Feature size that can be achieved using a typical screen printer is around 200 *um* ∼ 500 *um*, while the size of the square hydrocolloid patch under this study is 10 cm × 10 cm. Igreja-Dias model of IDC was selected and tuned to serve as an accurate model for IDCs printed on Hydrocolloids. The model was developed on the basis of a few assumptions that will define the parameter space of the model:

1. The periodical structure of fingers is infinite.
2. Length of fingers is infinite or much larger than the feature size of the IDC $l > 2({\rm{s}}+{\rm{w}})=\lambda$.

**Figure 6:**
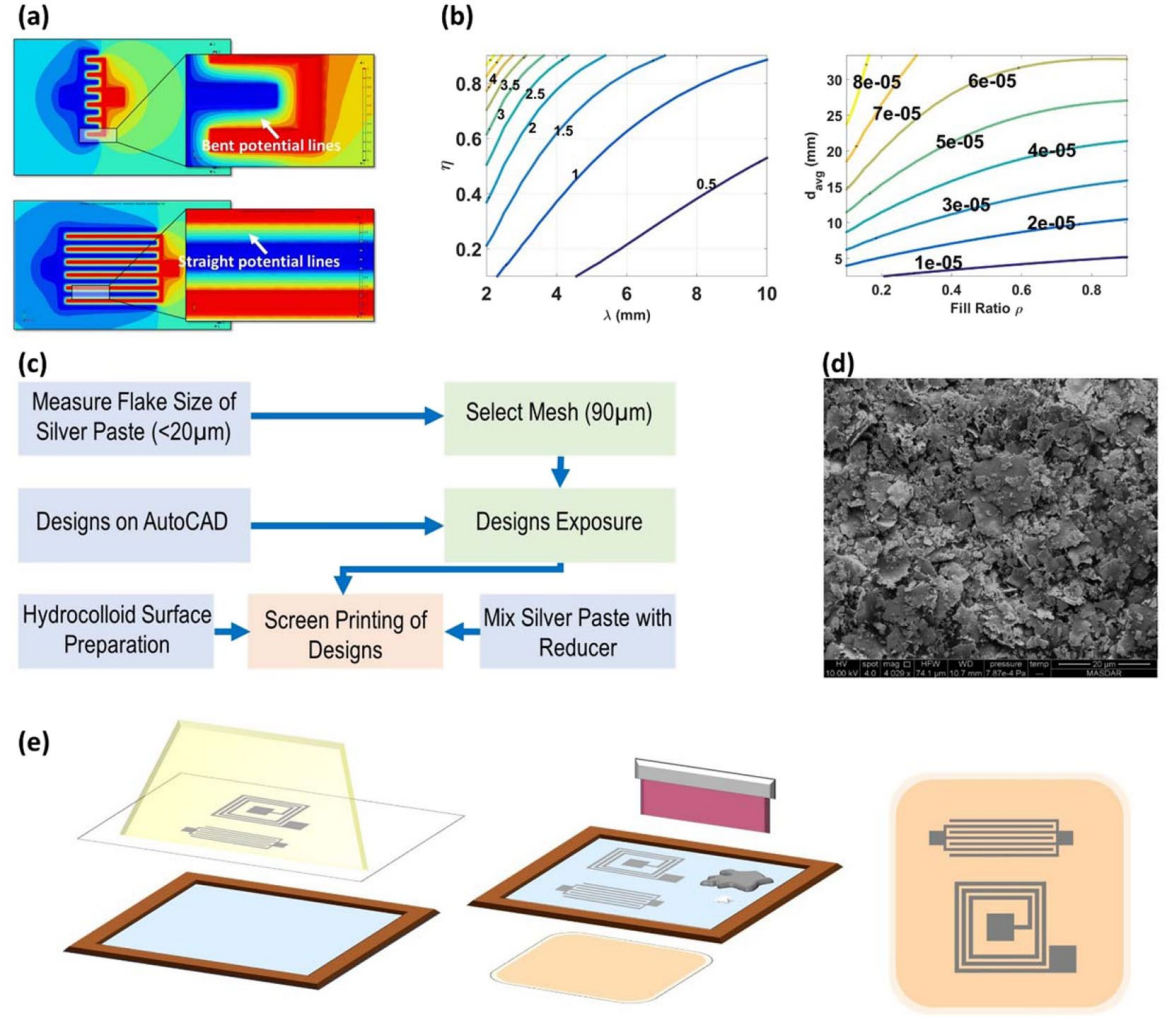
(**a**) Potential lines simulation in-between IDC fingers (**b**)Design contour plot for IDC (pF per cm^2^) and square spiral on a 10cmx10cm hydrocolloid. (**c**) Followed methodology for screen-printing (**d**) SEM micrograph of the Paron-910 silver paste flakes (**e**) Schematic explaining the process of screen-printing. Blue layer represents the emulsion. Passives designs are exposed to the mesh. Designs are then transferred to the mesh. Silver paste (silver liquid) and reducer (white liquid) are mixed, and the squeegee is used to mix and spread the mixture on the mesh and hence on the hydrocolloid beneath it. The designs are finally screen-printed on hydrocolloid.

These two assumptions indicate that potential lines are assumed to be perfectly flat in between the adjacent differently polarized electrode fingers as shown in Fig. 6a. Also, the model is developed for IDCs where *N* > 3. Taking these assumptions into consideration, the parameter space of the IDC model is:

6 $$0.1 < \eta =\frac{w}{s+w} < 0.9$$

7 $$2\,\lambda  < l < D$$

8 $$3 < N < {N}_{max}$$

where *N*_*max*_ can be calculated for a given *λ* and *η*, or in other words, for a given *s* and *w* given the equation below:

9 $${N}_{max} < \frac{D+s}{w+s}$$

Mohan’s corrected current sheet approximated model^20^ was selected after tuning to serve as an accurate model for square spirals printed on hydrocolloid. The current sheet approximation, though corrected, has a limitation on $\frac{s}{w}$ ratio, where it is not preferable to go beyond 3. Additionally, the model assumes the length of every straight conductor must be larger than its width. The smallest conductor length of a square spiral is equal to the inner diameter, *d*_*in*_. Considering these two assumptions, the model’s parameter space is:

10 $$m\cdot f < w < {d}_{in}$$

11 $$m\cdot f < s < 3w$$

12 $$m\cdot f < {d}_{in} < D-2(m\cdot f)$$

13 $$1 < N < {N}_{max}$$

where *N*_*max*_ is largest possible number of turns giving *s* and *w*. *N*_*max*_ can be realized as follows:

14 $${d}_{in}+2{N}_{max}(w+s) < D$$

15 $${N}_{max} < \frac{D-{d}_{in}}{2(w+s)}$$

Fig. 6b is an example design plot for a hydrocolloid generated using a 10 cm × 10 cm size. This contour plot studies the relationship between *λ* in *mm*, *η* and capacitance density in pF/mm^2^. It is clear that there are many realizations for the same value of capacitance density. However, there can be some factors that will make some realizations better than the others as mentioned in^19^. Capacitance density is used instead of capacitance to remove any area-related constrains. Similarly, it is possible to generate a design contour plot for the inductance in Henry as shown in Fig. 6b, where ${d}_{avg}=\frac{{d}_{in}+{d}_{out}}{2}$ and $\rho =\frac{{d}_{out}-{d}_{in}}{{d}_{out}+{d}_{in}}$. As the spiral inductor is square in shape, it is expected to allocate an area for the inductor such the L = W.

Finally, we demonstrated the use of medical hydrocolloids to serve as a platform for printed passives and electronics integration in the wearable electronics domain. Medical hydrocolloids are adhesive, flexible and medically approved. Hydrocolloids were physically and electrically characterized prior to printing. Several models for planar passives were explored and compared to COMSOL simulation results while considering the hydrocolloid properties. It was found that Igreja-Dias model^19^ after the proposed modification serve as an accurate model for printed IDC while the Current sheet approximation by Mohan^20^ was found to be an accurate model for square printed spiral inductors after including the proposed modifications. Proposed models gave mean percentage error of <6% as compared to measured fabricated samples. Two demonstration has been discussed in this work: IDC as a humidity sensor and spiral inductors for WPT. Lastly, limitations of the proposed models have been discussed.

## Methods

### Hydrocolloid surface characterization

Quanta 250 SEM by FEI was used to acquire several micrographs for the surface of the 3 M Tegaderm hydrocolloid. The surface micrographs were taken under specific conditions of beam energy, chamber pressure, and magnification. It can be noticed from Table 3 that the beam energy used is relatively low to prevent charges from accumulation as the hydrocolloid is a non-conductive material. Higher energy beam however was used for observing the flake size of the silver paste.

**Table 3:** SEM conditions for the hydrocolloid’s surface micrograph.

Figure	Beam Energy	Pressure	Magnification
Fig. 1c	2 kV	8.61 × 10^−4^ Pa	X 1033
Fig. 1d (left)	2 kV	6.83 × 10^−4^ Pa	X 58
Fig. 1d (right)	2 kV	4.55 × 10^−3^ Pa	X 54
Fig. 6d	10 kV	7.87 × 10^-4^ Pa	X4029

The cross-sectional micrograph needs more powerful SEM and thus used Nova NanoSEM 650 by FEI. This microscope provides higher magnification and enabled us to measure the thin layer of printed silver which was approximated to around 20 µm. The beam energy used in acquiring Fig. 2c is 1 kV at a magnification of x690.

### Passives fabrication

Fig. 6c shows the methodology followed in this work to fabricate the passives on hydrocolloids. All passives in this work were fabricated and printed on top of square 3 M Tegaderm Hydrocolloid dressings with a side length of 10 cm and thickness of around 1 mm. Prior to printing, hydrocolloids were laminated with a thin layer of Polyvinyl Acetate (PVA) which is dense enough to smoothen the un-even features of the surface while being sparse enough to maintain flexibility. Paron-910 silver paste which is distinguished by its low resistivity (15~50 mΩ/ϒ/mil) was utilized in screen-printing the passives on top of the prepared hydrocolloids. Silver paste flakes were characterized and their size was examined by Quanta250 SEM as shown in Fig. 6d. All passives were first designed on AutoCAD and exposed on a mesh with an opening of size 90 µm. A 1000 Watts mercury lamp was used to expose designs on the emulsion layer. Printing ink reducer by Sunglo is mixed with the silver paste to ease the process of printing and to reduce the amount of paste sticking on the mesh surface. The fabrication process is illustrated in Fig. 6e.

### Passives FEM simulations

**Figure 7:**
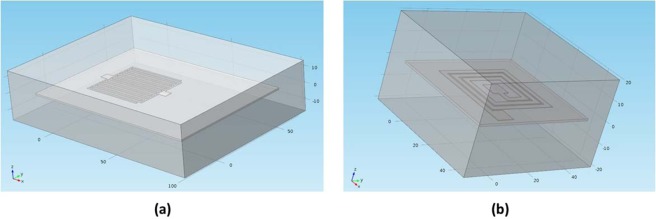
COMSOL geometry for (**a**) IDC simulations and (**b**) Spiral inductors simulations.

COMSOL simulations were conducted spanning wide range of IDC and spiral inductors geometrical configurations. These simulations were specifically adapted for the hydrocolloid’s characteristics. As shown in Fig. 7, the simulation environment consists of 3 geometrical elements: IDC/Spiral structure, the hydrocolloid itself and the surrounding air. Thickness of the hydrocolloid was set to 1.155 um as reported earlier. Simulation was run for 45 structures of IDCs and 45 structures of spirals which their geometrical parameters are listed in Tables (4, 5).

**Table 4:** Details of designed IDC sweeping wide range of parameters.

Property	Unit	min	step	max	Comments
Fingers	#	2	1	10	Width: 1 mm Spacing: 1 mm Length: 38 mm Thickness: 200 um
Finger Width	mm	0.5	0,5	4.5	Fingers: 6 Spacing: 1 mm Length: 38 mm Thickness: 200 um
Spacing	mm	1	1	9	Fingers: 6 Width: 1 mm Length: 38 mm Thickness: 200 um
Finger Length	mm	20	5	60	Fingers: 6 Width: 1 mm Spacing: 1mm Thickness: 200 um
Thickness	um	100	50	500	Fingers: 6 Width: 1 mm Spacing: 1 mm Length: 38 mm

**Table 5:** Details of designed spirals sweeping wide range of parameters.

Property	Unit	min	step	max	Comments
Number of Turns	#	3	1	11	Width: 1.2 mm Spacing: 0.8 mm Inner Space: 15.5 mm Thickness: 200 um
Spacing	mm	0.8	0.2	2.4	Width: 1.2 mm # of Turns: 3 Inner Space: 15.5 mm Thickness: 200 um
Width	mm	1	0.2	2.6	Spacing: 0.8 # of Turns: 3 Inner Space: 15.5 mm Thickness: 200 um
Inner Space	mm	13	2.5	35.5	Spacing: 0.8 # of Turns: 3 Width: 1.2mm Thickness: 200um
Thickness	um	13	2.5	35.5	Spacing: 0.8 # of Turns: 3 Width: 1.2mm Inner Space: 15.5 mm

It was found that both, the size of the air box and the thickness of the hydrocolloid have noticeable effect on the simulated capacitance and inductance. Hydrocolloid’s thickness was already measured under the Scanning Electronic Microscope (SEM) while the size of the air box was set to the minimum size possible after which the simulated capacitance/inductance doesn’t change. This is mainly because we aim to reduce the time of meshing and hence the computation time.

Materials were assigned to each of the three geometries: air, silver paste and hydrocolloid. Hydrocolloid is not available in the existing materials library, therefore, a new material was created. Electrical properties of the hydrocolloid were provided as part of the material’s definition. Table (6) below summarizes electrical properties of each of the materials that were used during simulations. The *Electric Currents (ec)* and the *Magnetic Field (mf)* submodules under the AC/DC module were used for running all IDCs and spirals simulations. Meshing is physics-defined and is usually set to Finer or Extra Fine depending on the structure. Fig. 7 shows COMSOL Multiphysics 3D geometry for both, the IDC and the spiral inductor.

**Table 6:** Summary of materials properties fed to COMSOL.

Property	Air	Silver Paste	Hydrocolloid
Conductivity (S/m)	1 × 10^−16^	126 × 10^3^	0.00414
Relative Permittivity	1.00054	1	4.7
Relative Permeability	1	1	1

### Electrical characterization of passives

Printed resistors, IDCs, and spiral inductors were characterized by E4980A LCR meter from Keysight to measure their resistance, capacitance, and inductance respectively. The tool was calibrated for open and short circuit tests, and measurements were conducted at a frequency of 2 MHz and the voltage level of 1 V peak to peak. LCR meter ports were hooked to two XYZ 500TRS micro-positioners from Quarter Research and needles of 3.5 µm tip diameter from Signatone for stable and accurate measurement. Needles tips were bent to avoid the destruction of the soft hydrocolloid surface and the fabricated passive. The permittivity measurement, shown in Fig. 1e, was performed on a hydrocolloid sample through *the self-parallel plate capacitance* in which we cover both sides of the hydrocolloid sample with silver paste forming a parallel plate capacitor. The capacitance of this parallel plate was measured as we scan the frequency up to 2 MHz and the permittivity was calculated using the dimensions of the sample, both the area (6.15 cm × 5.3 cm) and the thickness (1.155 mm). On the other hand, the resonance frequency measurement shown in Figs. 2d and 4c was obtained using E4990A Impedance Analyzer from Keysight. The tool was calibrated, and the same setup of probes and needles was used. Frequency scan was set between 500 kHz −83 MHz with a frequency step of 63.460 kHz. For reliability tests, passives where measured using Keysight 4294 A Impedance Analyzer having a frequency limitation upto 110 MHz.

### Wireless power transfer measurements

This experiment required a pair of inductors at a time for sending and receiving power. Four pairs of printed spiral inductors were fabricated, and their dimensions and geometry are shown in Fig. 4a. Both, the TX and the RX inductors were fixed perpendicular to the surface and parallel to each other. The TX inductor was connected to a Tektronix AFG3022B signal generator, and wires were stuck to the TX inductor pads by carbon tape which is intrinsically conductive. The RX inductor pads, on the other hand, were connected to DSOX3034A Digital Oscilloscope to monitor the wirelessly received signal. Each of the fabricated pairs of spiral inductors has been tested under inductive coupling mechanism using a power carrier of $f=5\,MHz$ and ${V}_{pp}=5\,V$ while being exactly parallel to each other. The distance between both inductors was varied between 5 mm–70 mm. The magnetic coupling experiment for L_1_ and L_2_ on the other hand were conducted after attaching a discrete capacitor of 22 pF to both the TX and RX inductors. To teste each LC tank pairs on their own resonance frequency, the resonance frequency was measured for each of the tanks (parallel connection) using E4990A Impedance Analyzer. The WPT experiment was conducted while the LC components are connected in series.

### Smart adhesive bandage measurements

The circuitry of the hydrocolloid-based smart bandage made use of two ICs: NE555P timer from Texas Instrument and 74LS393 dual 4-bit binary counter from Fairchild. Two 10 MΩ resistors and a 220 nF capacitor were also used for a successful operation of the timer. This circuit was powered by a 5 V from E3648A DC power supply by Agilent. This can be potentially replaced by a disc or flexible battery for easy integration and portability.

## Data Availability

No datasets were generated or analysed during the current study.
